# Anti-inflammatory effects of olanexidine gluconate on oral epithelial cells

**DOI:** 10.1186/s12903-019-0932-0

**Published:** 2019-11-08

**Authors:** Takuya Nii, Hiromichi Yumoto, Katsuhiko Hirota, Yoichiro Miyake

**Affiliations:** 1 Naruto Research Institute, Research and Development Center, Otsuka Pharmaceutical Factory, Inc, Takuya Nii, 115 Kuguhara, Tateiwa, Muya-cho, Naruto, Tokushima 772-8601 Japan; 2 Department of Periodontology and Endodontology, Institute of Biomedical Sciences, Tokushima University Graduate School, Tokushima, Japan; 3 Department of Oral Microbiology, Institute of Biomedical Sciences Tokushima University, Tokushima, Japan; 4 Present Address: Department of Medical Hygiene, Dental Hygiene Course, Kochi Gakuen College, Kochi, Japan; 5 Present Address: Department of Oral Health Sciences, Faculty of Health and Welfare, Tokushima Bunri University, Tokushima, Japan

**Keywords:** Olanexidine gluconate, *Porphyromonas gingivalis* lipopolysaccharide, Keratinocytes

## Abstract

**Background:**

Periodontitis is a biofilm-induced chronic inflammatory condition of the periodontium. Chemokines produced by the innate and acquired immune responses play a significant role in disease progression. Reducing biofilm formation and inflammatory response caused by chemokines is vital for preventing and treating periodontitis. Previously, we observed that treatment with 0.1% olanexidine gluconate (OLG) inhibited biofilm formation on saliva-coated hydroxyapatite. This study aimed to evaluate the anti-inflammatory effect of OLG on oral epithelial cells.

**Methods:**

We examined if OLG could inhibit the inflammatory responses caused by *Porphyromonas gingivalis* (*P. gingivalis*) lipopolysaccharide (LPS) and heat-killed *P. gingivalis* in immortalized human oral keratinocytes (RT7).

**Results:**

Treatment of RT7 with non-cytotoxic OLG concentrations significantly inhibited the production of inflammatory chemokines such as interleukin 8 (IL-8), C-C motif ligand 20 (CCL20), and growth-related oncogene protein-α (GRO-α), which are stimulated by *P. gingivalis* LPS in a concentration-dependent manner. Moreover, the inhibitory effects were observed regardless of the treatment time with *P. gingivalis* LPS (6, 12, or 24 h). OLG also significantly inhibited chemokine production stimulated by heat-killed *P. gingivalis*.

**Conclusions:**

The findings of this study suggest that treatment with OLG inhibits chronic inflammatory reactions in oral mucosal cells, such as periodontitis, caused by oral bacteria.

## Background

Periodontitis is a chronic inflammatory condition triggered by microbial biofilm formation in the periodontal pocket, which induces periodontal tissue destruction and leads to tooth loss [1]. The inflammatory response in periodontitis is initiated by the innate immune response and progresses by the acquired and innate immune responses. Both immune responses are regulated by many factors, including chemokines that are involved in the migration of phagocytic cells to the site of infection. Therefore, to prevent and treat periodontitis, biofilm formation and the inflammatory response to chemokines must be reduced.

The innate immune response is initiated by toll-like receptors (TLRs) when biofilm-forming bacteria, such as *Porphyromonas gingivalis* infect the periodontal tissue [2, 3]. TLRs recognize various bacterial products called pathogen-associated molecular patterns (PAMPs), including lipopolysaccharide (LPS). Both in vitro and in vivo studies have reported that TLRs are expressed in the human gingival epithelium [4–7]. TLRs, which are highly expressed in the epithelial cells of the periodontal pocket tissue, were reported to recognize PAMPs of *P. gingivalis* and participate in the signaling pathway that induces the production of chemokines, such as interleukin-8 (IL-8) [4, 5].

IL-8 is produced during the initial steps of the inflammatory response and plays a crucial role in the recruitment and activation of neutrophils, which are major contributors to tissue damage during inflammatory diseases [8–10]. In recent years, growth-related oncogene protein-α (GRO-α) has been recognized for its chemotactic activity on neutrophils [11]. In a three-dimensional co-culture model of gingival epithelial cells, the expression levels of GRO-α were elevated in response to increasing inflammatory reactions [12]. Apart from the innate responses, the acquired immune response involving the invasion of T-cells and B-cells is also known to be involved in periodontitis.

The role of T helper type 17 (Th17) cells in the acquired immune response was recently reported, and their presence in periodontal tissue was confirmed [13]. The chemokine C-C motif ligand 20 (CCL20) is known to regulate Th17 cell migration and infiltration [14] and its expression, particularly in the basal layer of inflamed gingival epithelial cells, has been previously reported [15]. Therefore, the inhibition of chemokines appears to be essential in the treatment and prevention of periodontitis.

**Fig. 1:**
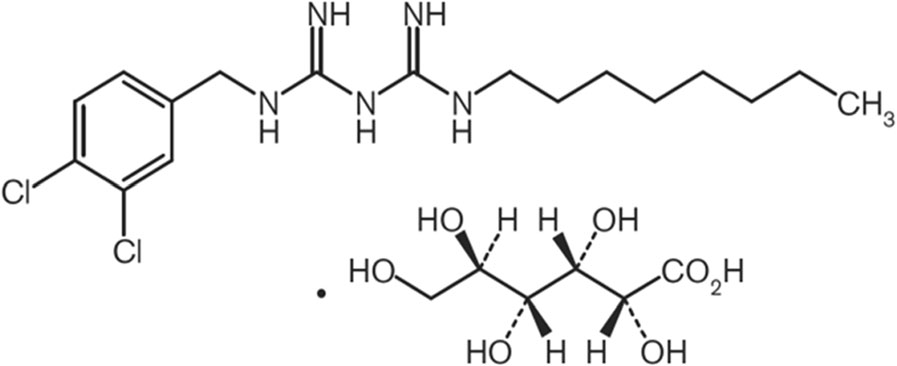
Chemical structure of Olanedine

Olanedine_®_ antiseptic solution 1.5% (Olanedine, Fig. 1) was launched in 2015 as an antiseptic for preoperative skin preparation. It contains olanexidine gluconate (OLG) [1-(3,4-dichlorobenzyl)-5-octylbiguanide gluconate] as an active ingredient. Olanedine is more effective bactericide than existing antiseptics in sites contaminated with blood [16] and acts against a broad spectrum of bacteria, including some that are drug-resistant [17, 18]. Besides, Olanedine has fast-acting and long-lasting bactericidal effects [16]. Based on these characteristics, OLG is considered an effective chemical plaque control agent in oral hygiene management.

In a previous study, we observed that 0.1% OLG inhibited the adherence of *Streptococcus mutans* on saliva-coated hydroxyapatite (HA) pellets. The results suggested that OLG covered the surface and reduced the hydrophobic interactions between *S. mutans* and saliva-coated HA-pellets, which form the salivary pellicle (unpublished). Since OLG can coat the surface and has a high binding affinity for LPS [17], we hypothesized that 0.1% OLG could inhibit the inflammatory response induced by bacteria or LPS stimulation, including the production of IL-8, GRO-α, and CCL20, in the oral mucosal epithelium. These anti-inflammatory effects might be valuable in preventing and treating periodontitis. Therefore, in this study, we examined if OLG inhibited the inflammatory reactions caused by *P. gingivalis* LPS or heat-killed *P. gingivalis* using immortalized human oral keratinocytes (RT7).

## Methods

### Cell lines and reagents

The 0.1% OLG formulation (Fig. 1) was composed of the solubilizing agent, polyoxyethylene polyoxypropylene glycol (POEPOPG), and the active ingredient olanexidine, and was prepared by Otsuka Pharmaceutical Factory, Inc. (Tokushima, Japan). Dilutions were prepared with phosphate buffered saline (PBS). The RT7 cell line, kindly provided by Dr. N. Kamata (Hiroshima University, Japan), was cultured in Keratinocyte-SFM (Thermo Fisher Scientific, MA, US) as described previously [19]. *P. gingivalis* strain ATCC 33277 was obtained from ATCC (Manassas, VA, US) and cultured in brain-heart infusion broth (Difco, Detroit, MI, US) supplemented with 0.5% yeast extract (Difco), hemin (10 μg/mL), and vitamin K (1 μg/mL) and harvested in the stationary phase. Bacterial numbers were determined spectrophotometrically using a standard curve adjusted with PBS (pH 7.4). Heat-killed *P. gingivalis* was prepared by heating a bacterial suspension (~ 1 × 10^9^ cells/mL) for 10 min at 65 °C. *P. gingivalis* LPS purified by a standard preparation is a TLR2 and TLR4-specific ligand and was commercially obtained from InvivoGen (San Diego, CA, US).

### Cytotoxicity test

RT7 were cultured to confluent monolayers in 24-well plates. After the aspiration of the medium, 0.2 mL of OLG at each concentration was added to the RT7 monolayer in each well and left to stand for 1 min at room temperature (RT). After the aspiration of OLG, fresh medium (0.5 mL/well) was added and RT7 monolayers were cultured further. After 6, 12, and 24 h of cultivation, total culture supernatants were collected to determine the concentration of lactate dehydrogenase (LDH) using the LDH cytotoxicity assay kit (Cayman Chemical Co., MI, US). At 24 h, the cell morphology of the cultures was observed using an inverted microscope (Nikon ECLIPSE TS100LED-F, Tokyo, Japan). RT7 monolayers, treated with 0.1% Triton X-100 for 10 min at RT, were used as a positive control in the cytotoxicity test.

### Measurement of inflammatory chemokines

We added fresh medium with 1 μg/mL *P. gingivalis* LPS (0.5 mL/well) to RT7 monolayers treated with 10 or 50 μg/mL OLG (1 min at RT). After 6, 12, and 24 h of incubation, total culture supernatants were collected to determine the concentration of IL-8, CCL20, and GRO-α using an ELISA kit (R&D systems, MN, US). To measure the cytokines stimulated with heat-killed *P. gingivalis*, fresh medium containing heat-killed *P. gingivalis* at a multiplicity of infection (MOI) of 10 were added to the RT7 monolayer treated with 50 μg/mL OLG (1 min at RT).

### Statistical analysis

All statistical analyses were performed using the unpaired Student’s *t*-test and GraphPad Prism (GraphPad Software, Inc. La Jolla, CA, USA). Differences were considered significant when the probability value was less than 5% (*p* < 0.05).

## Results

### Cytotoxicity of OLG on oral epithelial cells

**Fig. 2:**
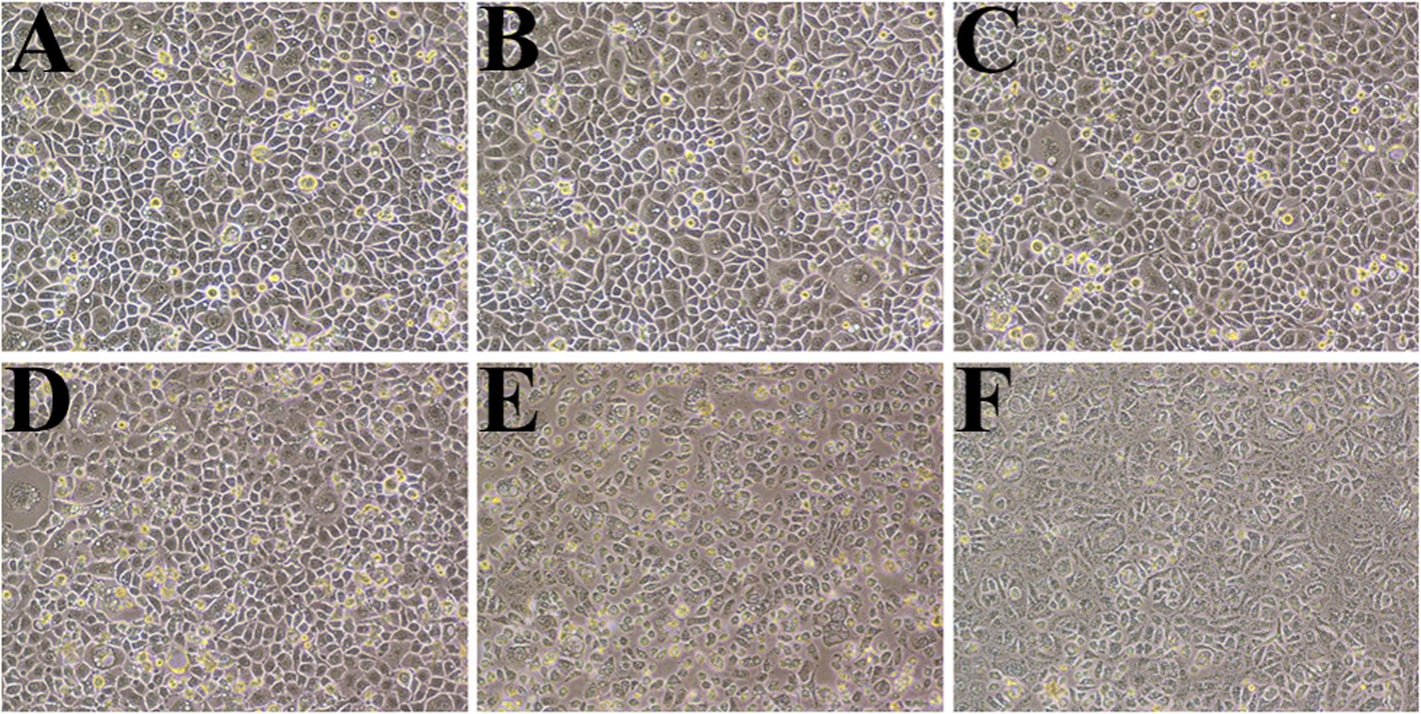
Morphology of RT7 monolayers 24 h after treatment (1 min) with OLG. Each panel shows a representative photograph of the different treatments. Cytotoxicity was not observed in the control (**a**) and after treatment with 10 μg/mL (**b**) and 50 μg/mL (**c**) OLG, while morphological changes were observed after treatment with 100 μg/mL (**d**), 250 μg/mL (**e**), and 500 μg/mL (**f**) OLG. OLG: olanexidine gluconate

**Fig. 3:**
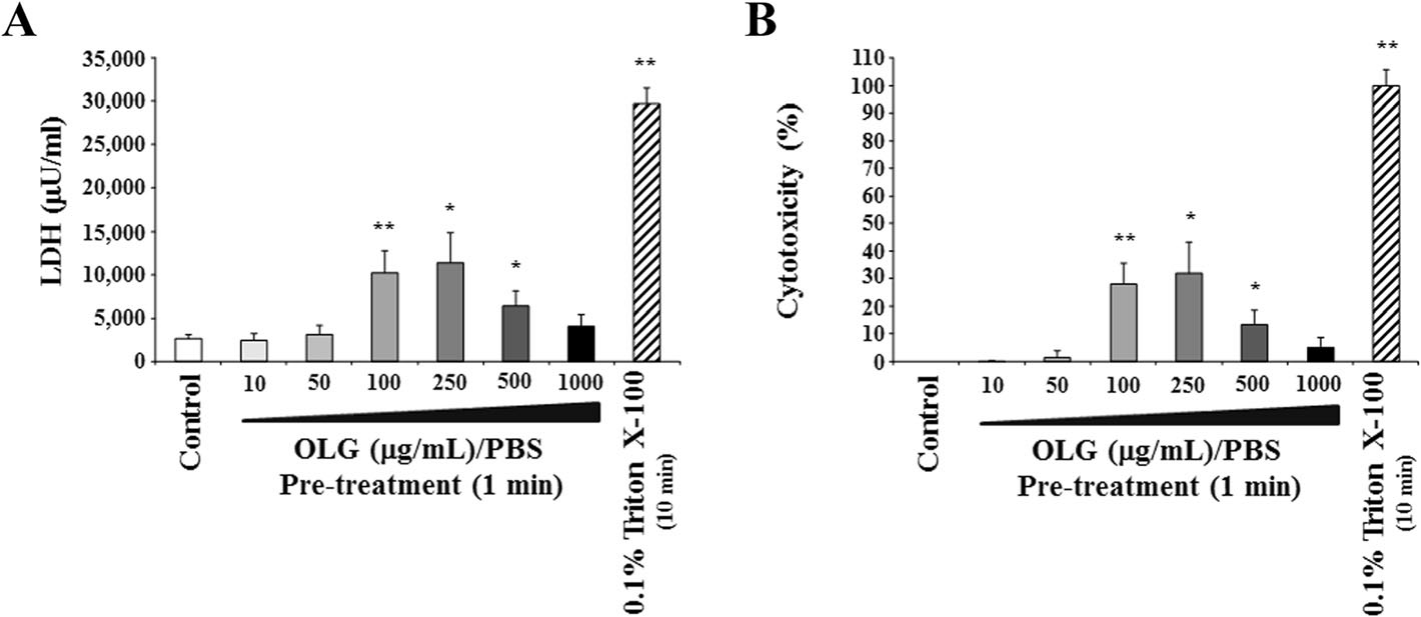
LDH measurement (**a**) and cytotoxicity (**b**) 24 h after treatment with OLG (1 min). Cytotoxicity was not observed after treatment with 10, 50, and 1000 μg/mL OLG when compared to the control. The values are expressed as the mean ± standard deviation of four replicate measurements. * (*p* < 0.05) and ** (*p* < 0.01) show significant differences when compared to the control. LDH: lactate dehydrogenase; OLG: olanexidine gluconate

**Fig. 4:**
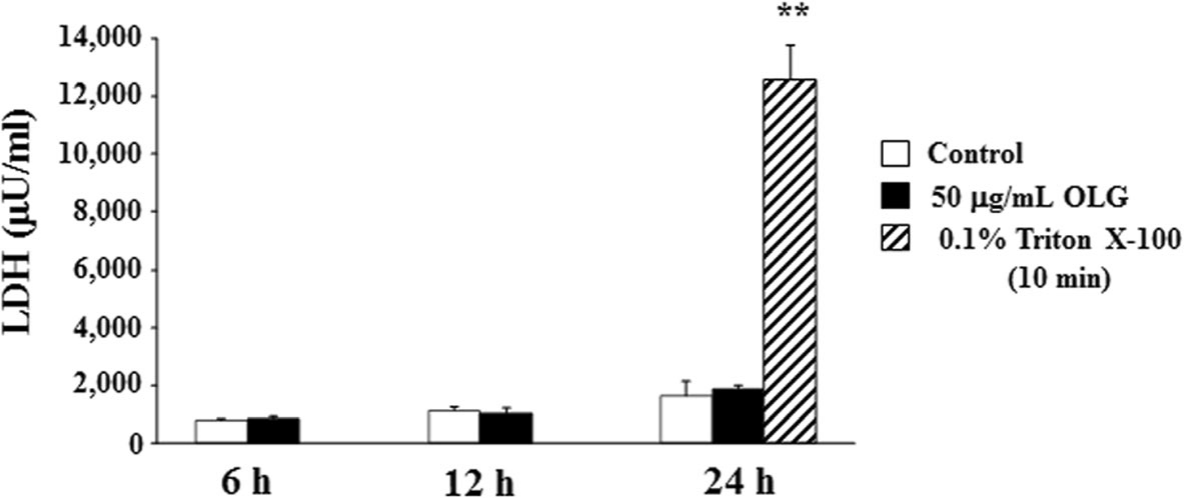
LDH measurement at different time points after 1 min treatment with 50 μg/mL OLG. Cytotoxicity was not observed at 6, 12, and 24 h. The values are expressed as the mean ± standard deviation of four replicate measurements. ** (*p* < 0.01) shows significant difference when compared to the control. LDH: lactate dehydrogenase; OLG: olanexidine gluconate

OLG concentrations less than or equal to 50 μg/mL showed no cytotoxic effects on the morphology of the cells (Fig. 2). The LDH concentrations corroborated this observation (Figs. 3 and 4). Upon morphological analysis, cytotoxicity was observed at concentrations higher than 100 μg/mL OLG; however, no LDH cytotoxicity was observed even at 1000 μg/mL OLG.

### Inhibitory effect of OLG on the production of chemokines IL-8, CCL20, and GRO-α

**Fig. 5:**
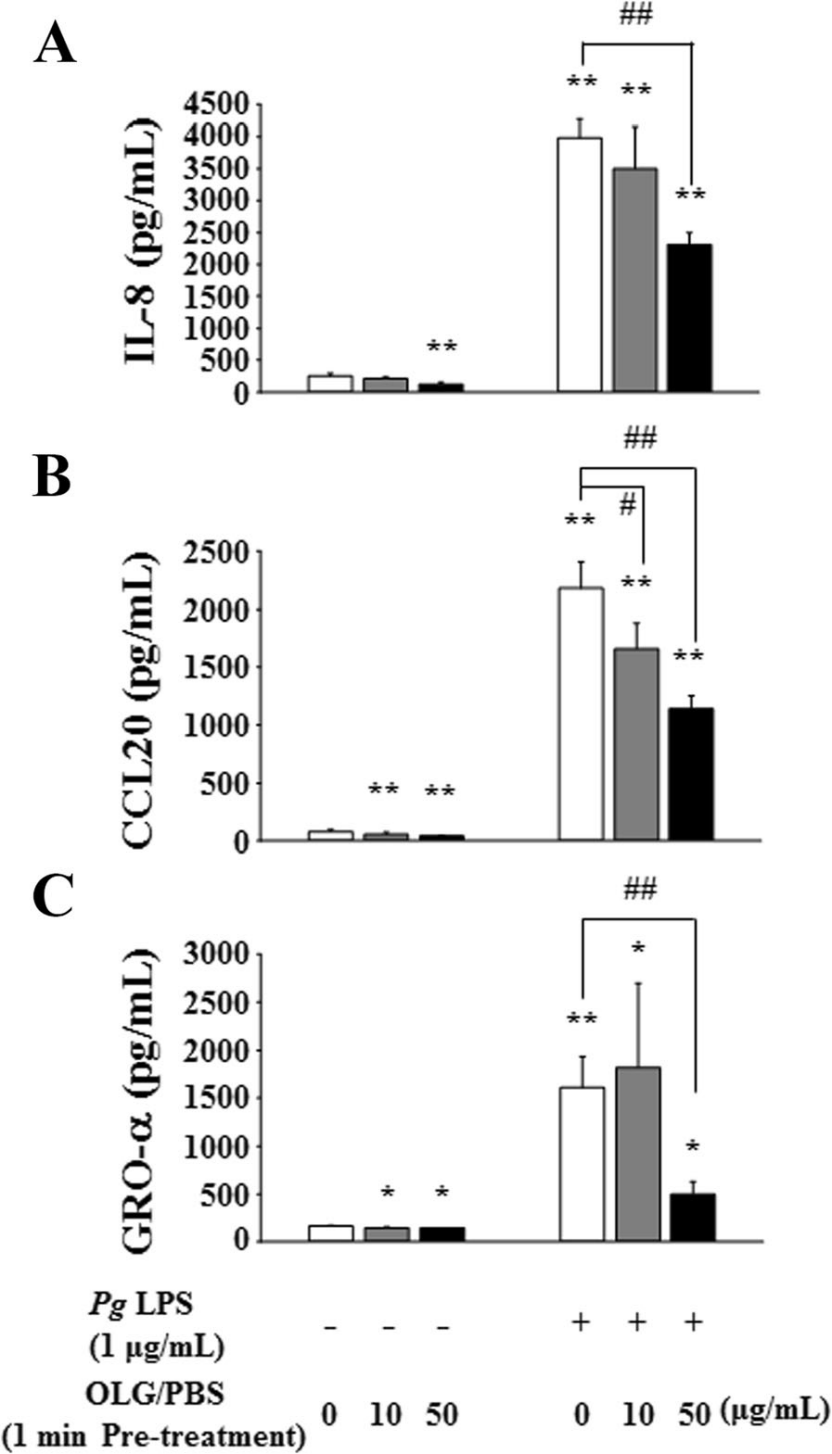
OLG inhibited chemokine production in RT7 stimulated by *P. gingivalis* LPS for 24 h. Treatment with 10 and 50 μg/mL OLG significantly inhibited the production of interleukin 8 (IL-8) (**a**) and C-C motif ligand 20 (CCL20) (**b**) in a concentration-dependent manner and treatment with 50 μg/mL OLG significantly inhibited the production of growth-related oncogene protein-α (GRO-α) (**c**). The values are expressed as the mean ± standard deviation of four replicate measurements. * (*p* < 0.05) and ** (*p* < 0.01) show significant differences when compared to the non-stimulated control. # (*p* < 0.05) and ## (*p* < 0.01) show significant differences when compared to the control stimulated by *P. gingivalis*. OLG: olanexidine gluconate; *Pg*: *P. gingivalis*; LPS: lipopolysaccharide

**Fig. 6:**
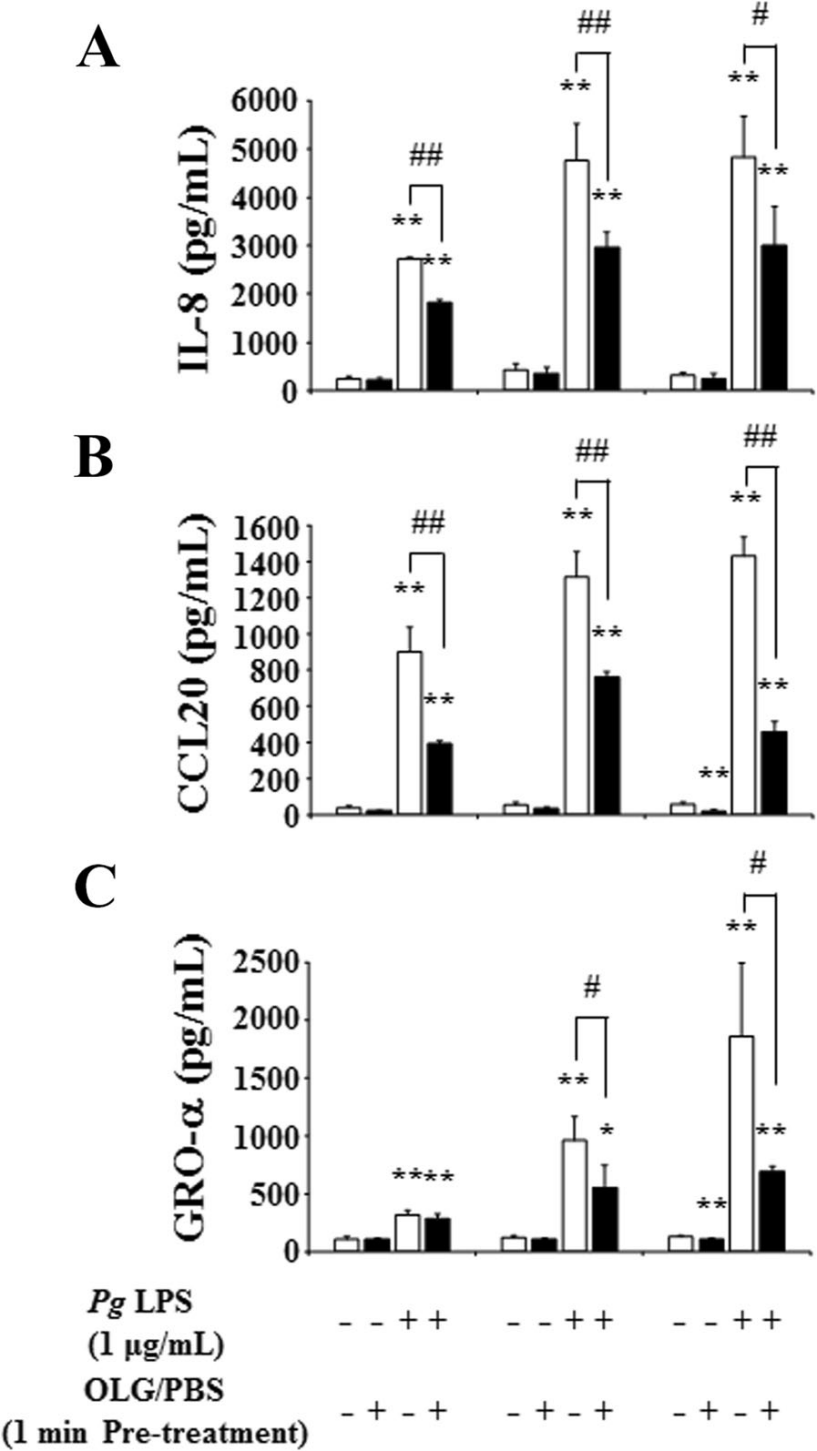
OLG inhibited chemokine production in RT7 stimulated by *P. gingivalis* LPS at each time point. Treatment with 50 μg/mL OLG significantly inhibited the production of interleukin 8 (IL-8) (**a**), C-C motif ligand 20 (CCL20) (**b**), and growth-related oncogene protein-α (GRO-α) (**c**) after 6, 12, and 24 h of stimulation with 1 μg/mL *P. gingivalis* LPS. The values are expressed as the mean ± standard deviation of four replicate measurements. * (*p* < 0.05) and ** (*p* < 0.01) show significant differences when compared to the non-stimulated control. # (*p* < 0.05) and ## (*p* < 0.01) show significant differences when compared to the control stimulated by *P. gingivalis*. OLG: olanexidine gluconate; *Pg*: *P. gingivalis*; LPS: lipopolysaccharide

OLG treatment significantly inhibited the production of IL-8, CCL20, and GRO-α after 24 h of stimulation by *P. gingivalis* LPS in a concentration-dependent manner (Fig. 5). Furthermore, treatment with 50 μg/mL OLG significantly inhibited the production of chemokines after stimulation by 1 μg/mL *P. gingivalis* LPS at all time points (6, 12, and 24 h) (Fig. 6).

**Fig. 7:**
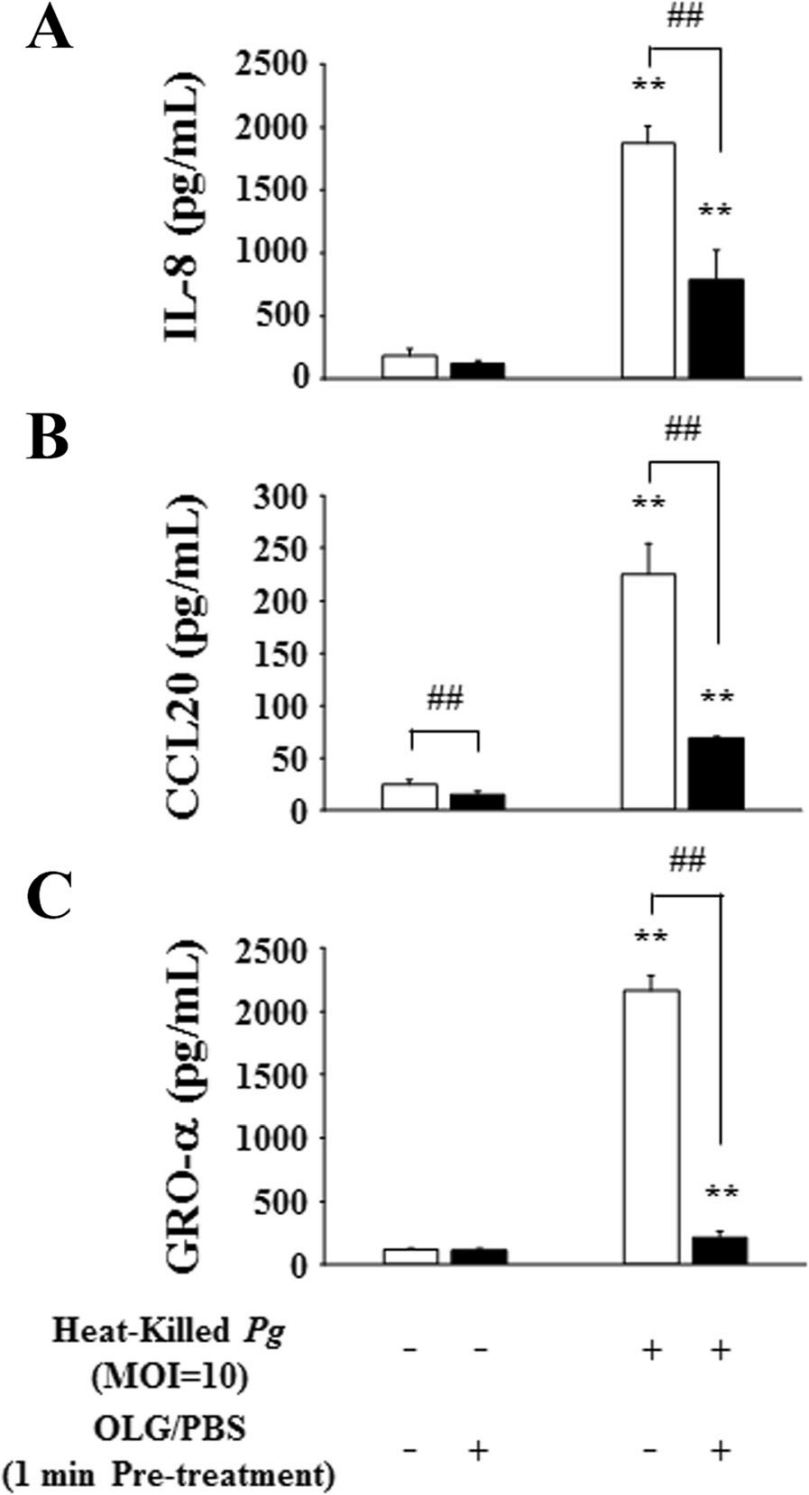
OLG inhibited chemokine production in RT7 stimulated by heat-killed *P. gingivalis*. Treatment with 50 μg/mL OLG significantly inhibited the production of interleukin 8 (IL-8) (**a**), C-C motif ligand 20 (CCL20) (**b**), and growth-related oncogene protein-α (GRO-α) (**c**) after 24 h of stimulation by heat-killed *P. gingivalis* (MOI = 10). The values are expressed as the mean ± standard deviation of four replicate measurements. ** (*p* < 0.01) shows significant differences when compared to the non-stimulated control. ## (*p* < 0.01) shows significant differences when compared to the control stimulated by heat-killed *P. gingivalis*. OLG: olanexidine gluconate; *Pg*: *P. gingivalis*; MOI: multiplicity of infection

As observed with *P. gingivalis* LPS, 50 μg/mL OLG treatment significantly inhibited the production of IL-8, CCL20, and GRO-α after stimulation for 24 h with heat-killed *P. gingivalis* (Fig. 7).

## Discussion

We examined the cytotoxic effects of OLG on RT7 and whether OLG inhibited the inflammatory responses stimulated by *P. gingivalis* LPS or heat-killed *P. gingivalis*. Microscopic observation of cell morphology and the LDH cytotoxicity assay showed that up to 50 μg/mL of OLG (1 min at RT) were non-cytotoxic. Inconsistencies among the results of morphological analysis and LDH cytotoxicity analysis at 1000 μg/mL OLG could be due to the mode of action of OLG, which causes protein denaturation as is common in other antiseptics.

Recent research indicated that OLG disrupts bacterial membrane integrity and denatures proteins at relatively high concentrations (≥160 μg/mL) [17]. High concentrations of OLG could aggregate RT7 cells by protein denaturation, which prevents the release of LDH. Therefore, the amount of LDH released at 500 and 1000 μg/mL of OLG would be less than that released at 250 μg/mL or lower concentrations. To validate this, we confirmed that the released LDH was detected in the discarded medium after 1 min treatment of OLG (data not shown). When we analyzed the effects of long-term exposure (8–24 h at RT) to OLG, we detected cytotoxicity at lower concentrations (≥5 μg/mL). However, the results of this study suggest that a short exposure period of approximately 1 min is sufficient for the antiseptic to have an adequate effect, and therefore it would not be necessary to use it in conditions that would cause cytotoxicity.

**Fig. 8:**
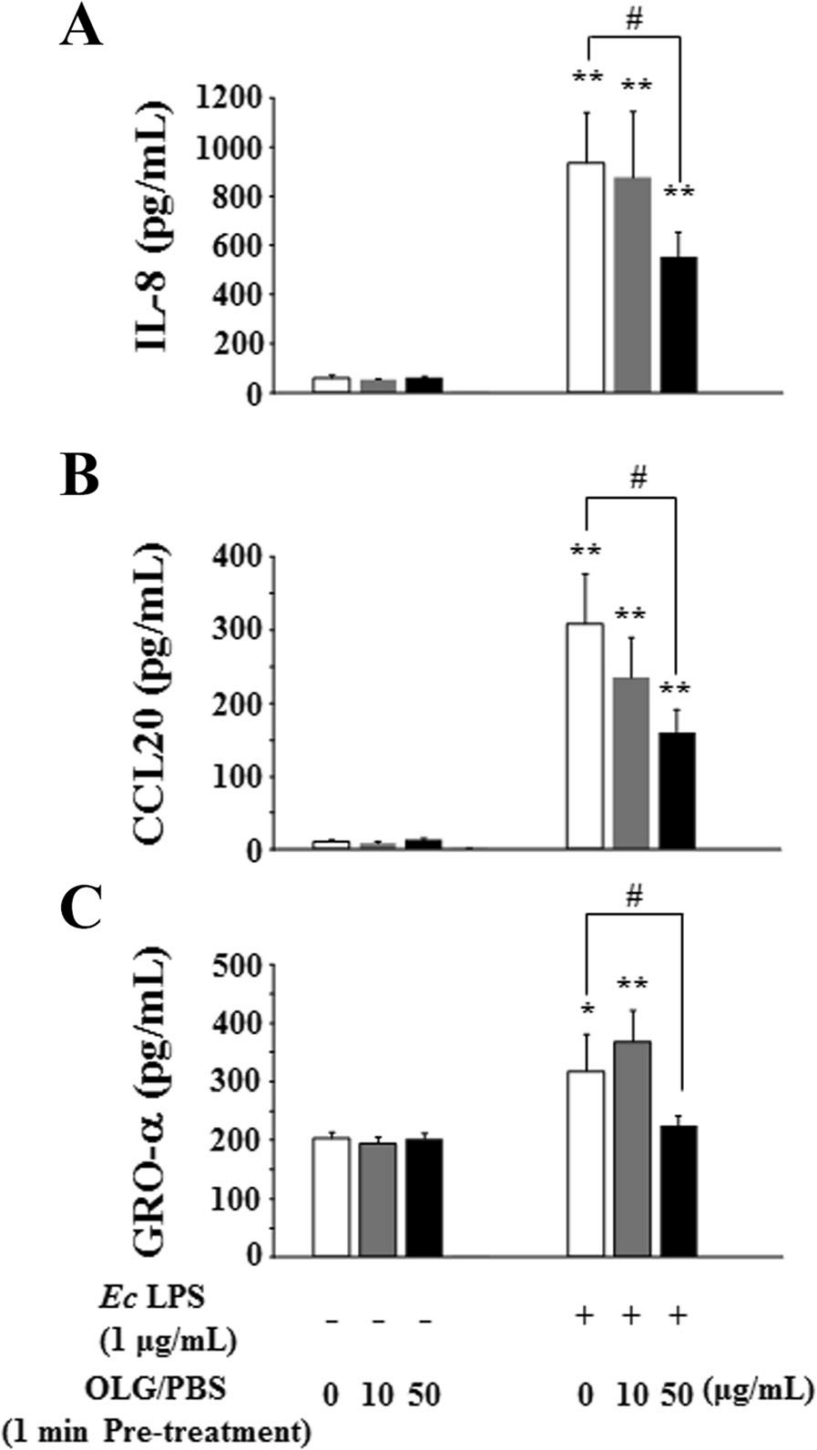
OLG inhibited chemokine production in RT7 stimulated by *E. coli* LPS for 24 h. Treatment with 10 and 50 μg/mL OLG significantly inhibited the production of interleukin 8 (IL-8) (**a**) and C-C motif ligand 20 (CCL20) (**b**) in a concentration-dependent manner and treatment with 50 μg/mL OLG significantly inhibited the production of growth-related oncogene protein-α (GRO-α) (**c**). The values are expressed as the mean ± standard deviation of four replicate measurements. * (*p* < 0.05) and ** (*p* < 0.01) indicate significant differences when compared to the non-stimulated control. # (*p* < 0.05) shows significant differences when compared to the control stimulated by *E. coli*. OLG: olanexidine gluconate; *Ec*: *E. coli*; LPS: lipopolysaccharide

Concerning the inflammatory responses, it was reported that oral and gingival epithelial cells stimulated with *P. gingivalis* LPS or heat-killed *P*. *gingivalis* had an increased mRNA expression and secretion of pro-inflammatory cytokines, including IL-8 [20–22]. In this study, the measurement of inflammatory chemokine levels showed that OLG at 10 and 50 μg/mL significantly inhibited the production of IL-8, CCL20, and GRO-α in RT7 cells stimulated with *P. gingivalis* LPS or heat-killed *P*. *gingivalis*. We performed the same experiment using *Escherichia coli* LPS (ultrapure LPS, *E. coli* 0111:B4, InvivoGen, San Diego, CA, US) to determine whether the anti-inflammatory effects of OLG were *P. gingivalis* LPS-specific. Results for *E. coli* LPS were the same as for *P. gingivalis* LPS; 10 and 50 μg/mL OLG significantly inhibited the production of IL-8, CCL20, and GRO-α in *E. coli* LPS-stimulated RT7 cells (Fig. 8). Considering past report [17], our results suggest that OLG inhibits LPS-induced inflammation in RT7 cells regardless of the bacterial species. In some experiments, the statistical data show minimal differences between untreated samples and those treated with OLG, even in cases of non-stimulation with *P. gingivalis* LPS or heat-killed *P*. *gingivalis*. However, we considered that there are no physiological differences based on the initial low expression levels of the different chemokines.

Chlorhexidine, one of the most effective antiseptics, was also reported to inhibit the inflammatory chemokine IL-8 at both mRNA and protein levels in oral epithelial cells. This effect conforms to the mechanism of action of chlorhexidine, which shows an immediate bactericidal activity, combined with prolonged bacteriostatic action, due to absorption on the active surface [23, 24]. Further experiments such as the analysis of the mRNA expression of TLR2 or 4 are needed to understand the mechanism underlying OLG anti-inflammatory action entirely. However, the results of this study and previous studies suggest that OLG exhibits inhibitory effects on the oral inflammatory response to LPS from *P. gingivalis*, a major pathogen causing periodontitis, by forming a protective layer over the gingival epithelial cells and through its bactericidal activity.

To fully understand the effects of utilizing 0.1% OLG in the clinical practice, we would need to perform in vivo studies. However, since chlorhexidine has been approved for the management of gingivitis, the use of 0.1% OLG as a new chemical plaque-control agent for oral hygiene management to prevent oral infections, including dental caries and periodontal diseases, should be expected in the future.

## Conclusion

OLG treatment inhibited the production of chemokines IL-8, CCL20, and GRO-α in oral epithelial cells stimulated with *P. gingivalis* LPS or heat-killed *P. gingivalis* in vitro.

## Data Availability

The datasets used and/or analyzed during the current study are available from the corresponding author on reasonable request.

## Abbreviations

CCL20: C-C motif ligand 20
GRO-α: Growth-related oncogene protein-α
HA: Hydroxyapatite
IL-8: Interleukin 8
LDH: Lactate dehydrogenase
LPS: Lipopolysaccharide
MOI: Multiplicity of infection
OLG: Olanexidine gluconate
*P. gingivalis*: *Porphyromonas gingivalis*
PAMPs: Pathogen-associated molecular patterns
PBS: Phosphate buffered saline
POEPOPG: Polyoxyethylene polyoxypropylene glycol
RT: Room temperature
RT7: immortalized human oral keratinocytes
Th17: T helper type 17
TLRs: Toll-like receptors
